# Climate Change, Genetics or Human Choice: Why Were the Shells of Mankind's Earliest Ornament Larger in the Pleistocene Than in the Holocene?

**DOI:** 10.1371/journal.pone.0000614

**Published:** 2007-07-18

**Authors:** Peter R. Teske, Isabelle Papadopoulos, Christopher D. McQuaid, Brent K. Newman, Nigel P. Barker

**Affiliations:** 1 Molecular Ecology and Systematics Group, Department of Botany, Rhodes University, Grahamstown, South Africa; 2 Department of Zoology and Entomology, Rhodes University, Grahamstown, South Africa; 3 Coastal and Marine Pollution, Natural Resources and the Environment, The Council for Scientific and Industrial Research, Congella, Durban, South Africa; Max Planck Institute for Evolutionary Anthropology, Germany

## Abstract

**Background:**

The southern African tick shell, *Nassarius kraussianus* (Dunker, 1846), has been identified as being the earliest known ornamental object used by human beings. Shell beads dated from ∼75,000 years ago (Pleistocene era) were found in a cave located on South Africa's south coast. Beads made from *N. kraussianus* shells have also been found in deposits in this region dating from the beginning of the Holocene era (<10,000 years ago). These younger shells were significantly smaller, a phenomenon that has been attributed to a change in human preference.

**Methodology/Principal Findings:**

We investigated two alternative hypotheses explaining the difference in shell size: a) *N. kraussianus* comprises at least two genetic lineages that differ in size; b) the difference in shell size is due to phenotypic plasticity and is a function of environmental conditions. To test these hypotheses, we first reconstructed the species' phylogeographic history, and second, we measured the shell sizes of extant individuals throughout South Africa. Although two genetic lineages were identified, the sharing of haplotypes between these suggests that there is no genetic basis for the size differences. Extant individuals from the cool temperate west coast had significantly larger shells than populations in the remainder of the country, suggesting that *N. kraussianus* grows to a larger size in colder water.

**Conclusion/Significance:**

The decrease in fossil shell size from Pleistocene to Holocene was likely due to increased temperatures as a result of climate change at the beginning of the present interglacial period. We hypothesise that the sizes of *N. kraussianus* fossil shells can therefore serve as indicators of the climatic conditions that were prevalent in a particular region at the time when they were deposited. Moreover, *N. kraussianus* could serve as a biomonitor to study the impacts of future climate change on coastal biota in southern Africa.

## Introduction

**Figure 1:**
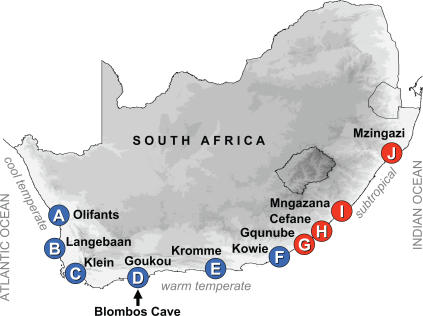
Sampling localities. Sampling localities (A–J) from which specimens of *Nassarius kraussianus* were collected. The sample sizes were: A = 350, B = 104, C = 132, D = 112, E = 47, F = 183, G = 83, H = 54, I = 71, J = 94. Blue circles indicate localities dominated by haplotypes present in the western range of the species' distribution, and red circles indicate sites where haplotypes of the eastern lineage were mostly found. The location of Blombos Cave (where *N. kraussianus* fossils were found that were worn as beads by human beings during the Middle and Late Stone Age) is indicated.

The southern African tick shell, *Nassarius kraussianus* (Dunker, 1846), is an estuarine and shallow water marine snail that has recently been identified as being the earliest known object to be used for ornamental purposes by human beings; shells found in Blombos Cave on the south coast of South Africa (Fig. 1) were estimated to have been worn as a necklace during the Middle Stone Age (MSA; ∼75 000 years ago [1], [2]). Fossils of *N. kraussianus* are absent from south-west African deposits of the Pliocene (1.8–5.3 million years ago; [3]), but by the late Pleistocene (∼120 000 years ago), the species had become an important component of the coastal fauna throughout much of southern Africa [4], [5]. Tick shells are also common in deposits from the Late Stone Age (LSA; beginning of the present interglacial, <10 000 years ago [2], [6]) and today are among the numerically dominant coastal gastropods in southern Africa [7]. Tick shells from Blombos Cave dated from the MSA were significantly larger than ornamental LSA shells from the same locality and from another fossil site in the region (Die Kelders), as well as from modern specimens collected in estuaries in the region (Goukou and Duiwenhoks) [2]. From this, D'Errico et al. [2] concluded that MSA humans had selected only exceptionally large shells for their necklaces, whereas during the LSA, shells were collected randomly. We question this interpretation for two reasons: Firstly, while several of the 29 MSA shells were more than 10 mm in length, none of the 2 098 LSA shells and none of the similarly sized 2 587 modern shells that were measured exceeded 10 mm. The effort of finding shells that exceeded 10 mm would thus have been exceptional during the LSA. Secondly, not all of the MSA shells were exceptionally large, but they rather comprised a size range from 6.83 to 10.42 mm. This could indicate that instead of a change in human preference, the sizes of shells in the region decreased. We tested two alternative hypotheses, both of which assume that shell sizes of *N. kraussianus* on South Africa's south coast were larger in the MSA than in the LSA: a) the size difference has a genetic basis and *N. kraussianus* comprises at least two distinct genetic units and b) the difference in shell size is due to phenotypic plasticity responding to changing environmental conditions.

### Genetic differentiation

The present-day geographical range of *N. kraussianus* spans three marine biogeographical provinces in southern Africa [7]: the cool-temperate west coast, the warm-temperate south coast, and the subtropical east coast (Fig. 1). Many marine organisms in the region are associated with more than one of these biogeographic provinces, but each province has its own combination of species [8]–[10]. Genetic data show that some coastal invertebrates with planktonic larvae or direct development that occur in more than one province comprise two or more genetically distinct regional lineages, with boundaries that coincide with those between the biogeographic provinces [11]–[17]. Given the wide distribution range of *N. kraussianus* and the fact that it disperses by means of planktonic larvae [18], it is possible that this species may comprise several regional phylogeographic units that differ in shell size, e.g. a large-bodied lineage adapted to cooler water temperature and a small-bodied lineage present in warmer water. Their distributions may have shifted as a result of climate change, with the small-bodied lineage having replaced the large-bodied lineage on the south coast. Alternatively, the larger lineage may have become extinct (e.g. during the last glacial maximum ∼15–25 000 years ago) and been replaced by the smaller lineage throughout its range (e.g. at the beginning of the present interglacial, <10 000 years ago). The two lineage-hypothesis is rejected if a) no genetically distinct lineages of *N. kraussianus* are found that differ in shell size and b) expansion events from south-eastern to south-western Africa evident in the genetic data do not significantly postdate the MSA.

### Phenotypic plasticity

Morphological differentiation in molluscs is often the result of phenotypic plasticity rather than genetics [19]. The fact that both homeotherms and poikilotherms grow larger at colder temperatures [20], [21] may account for a change in shell size as a result of an increase in water temperatures on the south coast from the MSA to the LSA. This hypothesis is rejected if tick shells in different biogeographic provinces do not differ in shell size.

## Methods

### Genetic analyses

A total of 1230 specimens of *Nassarius kraussianus* were collected from 10 South African estuaries/lagoons throughout the species' distribution range (Fig. 1). Ten specimens from each sampling locality were randomly selected for genetic analyses. Genomic DNA was isolated following the Chelex^®^ extraction protocol [22]. Partial mitochondrial *cytochrome oxidase c subunit I* gene (mtDNA *COI*) and *16S rDNA* sequences were obtained by means of the polymerase chain reaction (PCR) using universal primers [23], [24]. PCR reactions and sequencing followed previously published protocols [13], [25].

A minimum spanning network of mtDNA haplotypes was constructed using statistical parsimony [26] as implemented in the program tcs version 1.21 [27]. Gaps were treated as missing data. Relationships between groups of haplotypes (nested clades) and geography were established with the program geodis version 2.5 [28], and the latest version of the inference key for nested clade analyis [29] was used to infer the most likely evolutionary scenario that may have resulted in the observed phylogeographic patterns. As gene flow along the coast is essentially one-dimensional, along-coast distances between sampling localities were specified rather than the geographic coordinates.

To determine whether *N. kraussianus* underwent a range expansion, the mismatch distribution [30] of the mtDNA sequences was estimated under the spatial expansion model [31] in arlequin version 3.1 [32]. The time at which this event had taken place was determined by converting the expansion time parameter τ to time in years using the formula τ = 2ut, where u is the mutation rate per nucleotide per year multiplied by sequence length (i.e. number of nucleotides), and t is the time since population expansion in years. Ninety-five percent bootstrap confidence intervals of τ were calculated using 100 000 coalescent simulations in arlequin. The procedure was repeated five times to check for consistency of results. We also estimated the divergence time between two regional lineages of *N. kraussianus* identified by the nested clade analysis using the program im [33]. This program simultaneously estimates divergence time, time to most recent common ancestor, effective population sizes of the present-day lineages and their ancestor, the proportion of the ancestral population that has founded one of the descendant populations (to account for changes in population size), as well as pairwise migration rates, under the coalescent model [34]. We specified the HKY model [35] with an inheritance scalar of 0.25 for mitochondrial DNA. After a number of exploratory runs to determine suitable upper bounds for each model parameter, we used the following search strategy: -b500000–q1100–q2200–qa50–qu1–t2.5–m15–m210–fg–n20–g10.01–g22–k20–j8 (population1: western; population 2: eastern). To ensure consistency of results, 10 independent runs with random starting seeds and at least 2 million genealogical steps were performed. Final divergence time estimates were calculated based on the means of the five runs with the highest effective sample sizes (ESS). The value obtained for divergence time was converted to time in years using the formula t = *t*/u, where t is time in years, *t* is scaled divergence time and u is the mutation rate per site multiplied by sequence length.

A mutation rate to convert both τ and *t* into time in years was obtained as follows: for *COI*, we used an evolutionary rate of 1% per million years based on a marine gastropod group for which a good fossil record is available [36]. A rate for *16S rDNA* was determined using two sister species of marine gastropods for which published sequences of both *COI* and *16S rDNA* are available, namely *Conus brunneus* and *C. regius* [37]. *COI* and *16S rDNA* sequences of these differ by 13% and 6%, respectively. This corresponds to an evolutionary rate of 0.4% per million years for *16S rDNA*, or 0.7% per million years for the combined fragment, taking into account differences in sequence length.

### Morphological analyses

Shell lengths of specimens from all 10 estuaries/lagoons were measured as described previously [2]. Measurements were made using digital callipers and rounded to the nearest 0.05 mm. In most cases, the samples included a small number of juveniles and sub-adults (identified using the criteria in d'Errico et al. [2]). As the differences between adults and large sub-adults were not always obvious, we removed the smallest 25% of individuals from the data set of each population (following d'Errico et al. [2]) to eliminate the impacts of differences in recruitment between populations when comparing shell sizes of extant populations with the MSA shells. Ninety-five percent confidence intervals of the means were estimated based on 10 000 bootstrap pseudoreplications as implemented in PopTools version 2.6.3 [38].

## Results

### Genetic analyses

**Figure 2:**
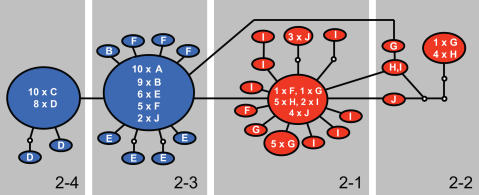
Network of *Nassarius kraussianus* haplotypes. A statistical parsimony haplotype network constructed from combined partial mtDNA *COI* and *16S rDNA* sequences of *N. kraussianus*. Haplotypes shown in blue were mostly found in the western portion of the species' geographical range (localities A–F; Fig. 1) and those in red were mostly found in the eastern portion of its range (localities G–J). Haplotypes are represented as ovals, with sizes being proportional to a haplotype's frequency. Letters within ovals indicate in which sampling localities a particular haplotype was found and correspond to the letters in Fig. 1. Small white circles are interior node haplotypes not present in the samples. Grey areas around groups of haplotypes depict two-step nested clades.

Consensus sequences of 600 and 483 nucleotides were obtained for *COI* and *16S rDNA*, respectively. All unique sequences generated in this study were submitted to GenBank (accession numbers DQ456981–DQ456995 and EF636006–EF636023). A total of 28 mitochondrial haplotypes were identified. A haplotype network constructed from these comprises two regionally confined units that are both characterised by a star-like pattern (Fig. 2), indicating population expansion [39]. Sixteen of the haplotypes were mostly present on the south-east coast (shown in red), including the basal haplotype of the network. Twelve haplotypes occurred primarily on the south-west coast (shown in blue), with only the basal haplotype of clade 2-3 being present but rare on the south-east coast. Significant genetic structure was found between clade 2-1 (which includes the basal haplotype of *N. kraussianus*) and clade 2-3 (which comprises younger haplotypes). Using nested clade analysis [29], the evolutionary scenario inferred for these clades was a contiguous range expansion from south-east to south-west (inference chain: 1-2-11-12-No).

The mismatch distribution of the *N. kraussianus* sequences did not depart from the expectations of the spatial expansion model (*SSD* = 0.003, *P* = 0.2), thus supporting the result from the nested clade analysis. A spatial expansion time parameter τ of 1.35 (95% C.I. = 0.94–2.72) was found, and the scaled divergence time *t* calculated for the divergence of the south-eastern and south-western lineages was 0.47 (95% C.I. = 0.26–1.21). Using a rate of 0.7% per million years for the combined *COI* and *16S rDNA* fragments, it was estimated that the range expansion from south-east to south-west took place ∼89 000 years ago (95% C.I. = 62–179 000 years ago), and that the two regional lineages then diverged ∼62 000 years ago (95% C.I. = 34–160 000 years ago).

### Morphological analyses

**Figure 3:**
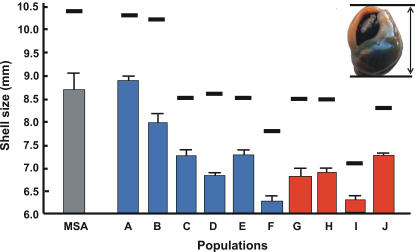
Shell sizes of *Nassarius kraussianus.* Shell sizes of the Middle Stone Age (MSA) specimens of *N. kraussianus* from Blombos Cave [2] and the largest 75% of the shells of each of ten extant South African populations (A–J). Vertical bars represent means and whiskers are upper 95% confidence limits. Horizontal lines represent the shell size of the largest individual from each population. The insert shows how shell size was measured.

Shells of extant populations from the west coast (Olifants Estuary and Langebaan Lagoon) were significantly larger than those from the south and east coasts, and individuals whose shells exceeded 10 mm in length were only found in these two populations. The largest 75% of the shells from the Olifants Estuary population were not significantly different in size from the MSA fossil shells (Fig. 3).

## Discussion

### Genetic analyses

In southern Africa, most of the examined coastal invertebrate species that, like *Nassarius kraussianus*, disperse by means of planktonic larvae, show considerable genetic differentiation across a transition area between the warm temperate and subtropical coastal regions in south-eastern Africa [11], [13], [14], [17]. Phylogeographic discontinuity in this region is also evident in *N. kraussianus*, but in contrast to other invertebrates studied to date, this differentiation is supported by comparatively few nucleotide differences, indicating that this species became established along the south-west coast relatively recently. The time estimates of demographic events in the evolutionary history of *N. kraussianus* suggest that these are likely to have been linked to climatic changes in the region. Fossil data indicate that warmer conditions during the last interglacial (∼80–130 000 years ago) enabled estuarine-lagoonal molluscs that are presently confined to the tropical east coast to extend their ranges to south-western Africa [4]. As *N. kraussianus* is also commonly found in deposits from this time and the range expansion estimate falls into this time (∼89 000 years ago), it is likely that this event took place as a result of elevated sea temperatures. In contrast to other gastropods, cooler temperatures after the last interglacial did not eliminate the species from south-western Africa. Confidence intervals of time estimates for the range expansion and subsequent divergence of the two lineages are wide. Nonetheless, the estimates do not significantly postdate the time when MSA shells were deposited at Blombos Cave (∼75 000 years ago), and they significantly predate the LSA. This suggests that there is little support for the hypothesis that a now extinct larger-bodied lineage may have been present on the south coast during the MSA and that it was subsequently replaced by a smaller-bodied lineage from the east. This conclusion is further strengthened by the fact that snails present on the cool-temperate west coast can attain sizes similar to those of the MSA individuals from the south coast, and that their mtDNA sequences are identical to individuals found as far east as the subtropical Mzingazi Estuary (Fig. 1).

### Morphological analyses

The rejection of the hypotheses that *Nassarius kraussianus* comprises or formerly comprised multiple regional genetic lineages that differ morphologically, suggests that shell size instead depends on environmental conditions. Body size in ectotherms has often been reported to be affected by temperature, with many species growing larger at lower temperatures [40]–[43]. The observed decrease in shell size in *N. kraussianus* between the two west coast populations and the remaining populations may thus be linked to sea surface temperatures, although microhabitat conditions are also likely to be important (the samples with the smallest shell sizes [localities F and I] were collected in shallow, vegetation-rich side arms of estuaries, where temperatures were higher than in the main channel). A decrease in shell size from west to east has also been reported in the South African rocky shore limpet *Patella granularis* [11]. Fossil shell sizes of marine gastropods in Chile showed marked increases during periods of intensified cold water upwelling throughout the Pleistocene and Holocene [44]. In South Africa, temperatures on the south and east coasts were cooler between ∼50–80 000 years ago than at present [45]. The fact that the shell sizes of *N. kraussianus* from the vicinity of the Goukou Estuary were similar to those of the present-day populations on the west coast suggests that similarly cooler environmental conditions were on the south coast during the MSA. We consider this the most parsimonious solution explaining the decrease in fossil shell size, and hypothesise that the sizes of *N. kraussianus* fossil shells can thus provide information on climatic conditions prevalent at the time during which they were deposited. The hypothesis that tick shells grow to a larger size in colder water could be further investigated by raising snails, collected at the same locality, at different temperatures in the laboratory. A confirmation of this would suggest that *N. kraussianus* could be a suitable biomonitor to study the effects of global climate change on the coastal biota of southern Africa, particularly because unlike most other southern African coastal invertebrates for which phylogeographic data are available, there is no indication of cryptic speciation in this species.

## Supporting Information

Figure S1 Plots of IM posterior probability distributions. Marginal posterior probability distributions for three parameter estimates from one of five IM runs scaled by the neutral mutation rate, including the population size parameter θ calculated for the southwestern lineage (θ^1^), southeastern lineage (θ^2^), and the ancestral lineage prior to divergence (θ^A^), time since population divergence (*t*) and migration rates (m^1^ = southwestern to southeastern; m^2^ = southeastern to southwestern). Posterior probabilities are shown on the y-axes.
(0.60 MB TIF)
