# Supplementary figures and images for: Climate Change, Genetics or Human Choice: Why Were the Shells of Mankind's Earliest Ornament Larger in the Pleistocene Than in the Holocene?
Source: PLoS One. 2007 Jul 18;2(7):e614. doi: 10.1371/journal.pone.0000614 (PMC1913204; doi:10.1371/journal.pone.0000614)

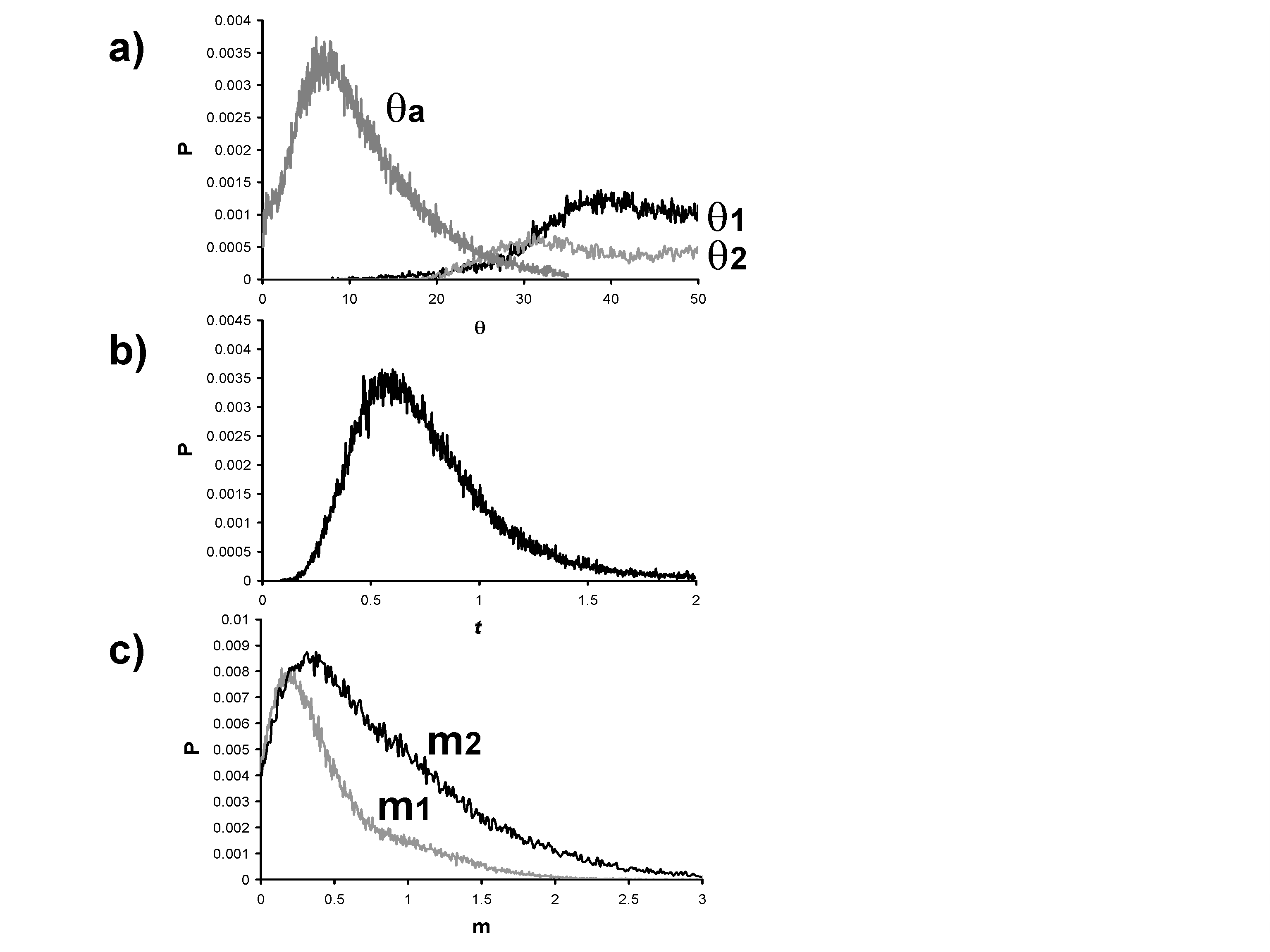

Supplement: Figure S1 — Plots of IM posterior probability distributions. Marginal posterior probability distributions for three parameter estimates from one of five IM runs scaled by the neutral mutation rate, including the population size parameter θ calculated for the southwestern lineage (θ1), southeastern lineage (θ2), and the ancestral lineage prior to divergence (θA), time since population divergence (t) and migration rates (m1 = southwestern to southeastern; m2 = southeastern to southwestern). Posterior probabilities are shown on the y-axes. (0.60 MB TIF) [file pone.0000614.s001.tif]
